# Serial kinematic analysis using inertial measurement units in growing dogs at risk of hip dysplasia

**DOI:** 10.1016/j.vas.2024.100385

**Published:** 2024-07-30

**Authors:** P. Blättler, M. Altermatt, M. Röhrich, N. Grütter

**Affiliations:** a Orthovet, Fasanenstrasse 13, CH-4402 Frenkendorf, Switzerland; b Institute for Medical Engineering and Medical Informatics, School of Life Sciences, University of Applied Sciences Northwestern Switzerland, Hofackerstrasse 30, Muttenz CH-4132, Switzerland; c HSEF s.r.o., Druzstevni 84, 691 81 Brezi, Czech Republic

**Keywords:** Inertial measurement unit, Canine gait, Movement analysis, Gait characteristics

## Abstract

In this study, 54 dogs were examined at regular intervals from 12 weeks to 15 months of age using a gait analysis system based on inertial measurement sensors. At the end of the study, the dogs were examined for hip dysplasia (HD) and elbow dysplasia (ED) under sedation and officially classified at the Dysplasia Commission in Zurich. Gait parameters which are characteristic for the gait pattern of dogs, were calculated according to recent publications. These parameters were analysed for variance throughout the entire study period and assigned to healthy dogs and those suffering from HD. The findings of the study show that dogs suffering from HD exhibit a more unsteady gait pattern, e.g. higher variance, as they grow.

## 1 Introduction

Hip dysplasia (HD) is a prevalent condition in dogs, affecting approximately 42 % of canine in Switzerland (Flückiger et al., 1999). It is regarded as a polygenic, multifactorial disease influenced by various factors such as nutrition, growth rate, and exercise (Boiocchi et al., 2013; Carr et al., 2016; Flückiger et al., 1999). Some authors also consider HD a biomechanical condition, emphasizing genetic predispositions along with factors like height, weight, and growth intensity as primary triggers (Comhaire & Snaps, 2008; Coros et al., 2011; Flückiger et al., 1999; Fortrie et al., 2015). Despite its prevalence, there is a notable gap in kinematic studies focusing on the movement of growing dogs, especially in the context of HD.

In the context of canine growth phases, the gait patterns of puppies undergo notable transformations due to rapid increase in weight between 12 and 24 weeks of age. This period is characterized by open growth plates, ongoing central nervous system (CNS) development, and immature musculature, contributing to an unstable gait (Carr et al., 2016). Additionally, exuberant play often results in musculoskeletal injuries and related disorders. Gait assessment and lameness diagnostics in small animal practices pose significant challenges, particularly in cases of intermittent or load-dependent lameness and gait alterations marked by relieving postures or incorrect limb loading.

Inertial measurement unit (IMU) sensors have emerged as valuable tools for detecting changes in the gait of dogs. IMUs measure acceleration, angular velocity, and sometimes magnetic field strength, providing comprehensive data on kinematic features. These sensors can capture movement patterns, helping in the early detection and classification of orthopedic diseases such as HD. Previous studies have demonstrated the utility of IMUs in various applications, yet a comprehensive classification of individual dogs' orthopedic diseases from a medical standpoint remains pending. The potential of IMUs to classify diseases through advanced methods like deep learning models, which can autonomously extract optimal features from raw spatiotemporal gait data, is promising (Taghavirazavizadeh et al., 2023).

This study aims to address the gap in literature by documenting the kinematic progression of different breeds from 12 weeks to 15 months of age. Our objective is to identify potential differences in the development of kinematic features between healthy dogs and those predisposed to HD. We hypothesize that dogs afflicted with HD exhibit a higher variance in movement compared to their healthy counterparts. This variance may be noticeable in stance and swing phases, range of motion, extension and flexion of movement, and mechanical symmetries, visually observable through indicators such as dogs running closely in the hindquarters, limping, or adopting a bow-legged hind leg position.

## 2 Material and method

### 2.1 Data generation

To verify this thesis, a 42-month study was initiated at the beginning of 2019 to investigate the kinematics of puppies from 12 weeks to fifteen months of age. Three breeds, comprising 37 Australian Shepherds, 11 Golden Retrievers and 6 Rottweilers, were selected for the study. The subjects were chosen out of a pool of available dogs due to the breed specific susceptibility to develop hip dysplasia. Between 12 and 28 weeks of age, the puppies underwent monthly routine examination and movement was measured using Motion Tracking (MTw) sensors. MTw's are miniature inertial measurement units (IMU) containing 3D linear accelerometers, 3D rates gyroscopes, 3D magnetometers. After 28 weeks of age, the intervals were extended to every 2 months and at the age of 15 months, the final orthopaedic examination was conducted, including BMI and movement diagnostics. During this last appointment, an official HD/ED X-ray was performed followed by evaluation through the HD Commission of the Zurich Veterinary Hospital.

#### 2.1.1 Inertial measurement units

**Fig. 1:**
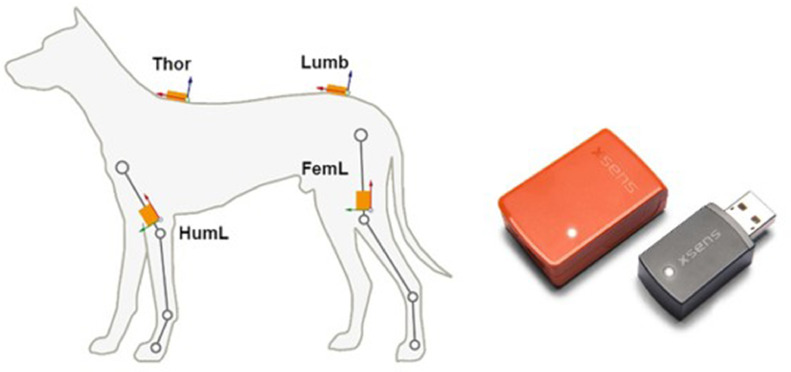
Left, schematic representation of the mobile gait analysis system with the positions of the IMU on the canine subject. Right: Inertial measurement unit from Movella Xsens used in the gait analysis with the associated receiving dongle.

The puppies’ movement was measured using IMU sensors with high measurement fidelity and sampling rate (Table 1). IMU's provides 3D angular velocity using rate gyroscopes, 3D acceleration using accelerometers supported with 3D magnetometers. IMU's are immune to magnetic disturbance. The IMU's were attached to the subject by a mobile gait analysis system which is described in the work of (Altermatt et al., 2023). A schematic representation of the gait analysis system and an illustration of a single IMU can be seen in Fig. 1.

**Table 1:** Orientation and tracker parameters.

Orientation & tracker parameters	
Dynamic accuracy (roll/pitch):	0.75 deg RMS
Static accuracy (roll/pitch):	0.5 deg RMS
Dynamic accuracy (heading):	1.5 deg RMS
Static accuracy (heading):	1 deg RMS
Tracker Components Dimensions:	3 axes
Full scale - gyroscope/ accelerometer/ magnetometer:	± 2000 deg/s / ± 160 m/s2 /± 1.9 Gauss
Magnetic imunity	Yes

According to former studies (Marreiros et al., 2017; Mobbs et al., 2022; Park & Yoon, 2021; Prasanth et al., 2021; Yoon et al., 2024) our findings indicate accuracy of IMU systems for detection of traditional metrics as well as the demonstrated clinical relevance of IMU measures.

#### 2.1.2 Feature detection

The data from the IMU sensors were analysed using an algorithm according to Altermatt et al. (2023). Their analysis yielded features characteristic of the gait pattern of dogs. These features describe the gait characteristics of the dogs on the day of the examination. An orthopedic veterinary surgeon selected a shortlist of specific kinematic gait parameters that may indicate the gait changes for further detailed analysis which we processed in the study.

Due to the measurement procedures adopted by the veterinary practice, up to 3 consecutive gait analyses were generated per dog on one day. The gait analysis is conducted by walking the subject on a leash in a straight line for 20 m. The dog is guided by the respective owner walking at normal pace as used on everyday walks. If the dogs were distracted by environmental influences, the procedure is repeated. To enable subsequent analysis, the mean values of the features generated on the same examination day were calculated. Furthermore, the data of the individual dogs were checked for the number of examination days. Any dog with fewer than 4 measurement days after pre-processing of the data was excluded from the analysis. As a result, a total of 44 dogs remained to be analysed, 14 of them diagnosed with HD by the HD committee of an independent veterinary hospital.

### 2.2 Preprocessing

The analysis and preprocessing were realised using Python (version 3.9.7) in the development environment Pycharm Community (version 2021.3.3). The libraries of Pandas (1.5.1), NumPy (1.22.3) and SciPy (1.8.0) were used.

Extensive preprocessing was carried out prior to the data analysis. This involved iteratively visualising the gait feature in dependency to the age of the dogs on the day of the measurement. A feature is defined as a characteristic describing the gait according to Altermatt et al. (2023). If implausible values were identified in the resultant plots, the entire data series for the respective day of the dog in question was subsequently excluded from the analysis. The definition of “implausible values” was based on the experience of a veterinary surgeon and includes for example detected step lengths of 2 m, velocities of 20 km/h etc. It should be noted that the generation of the anomalous values has its origin in the characteristic of the feature creation by the software and mainly concerns frequencies recorded twice.

The exclusion criteria of the features are listed in Table 2.

**Table 2:** Exclusion criteria for gait features.

**Feature**	**Exclusion criterion**
Energies	> 200 ≤ 0
Step lengths	> 0.6 m ≤ 0.1 m
Withers	> 0.6 m ≤ 0.15 m
Age	>450d

### 2.3 Outcome measure and analysis

**Fig. 2:**
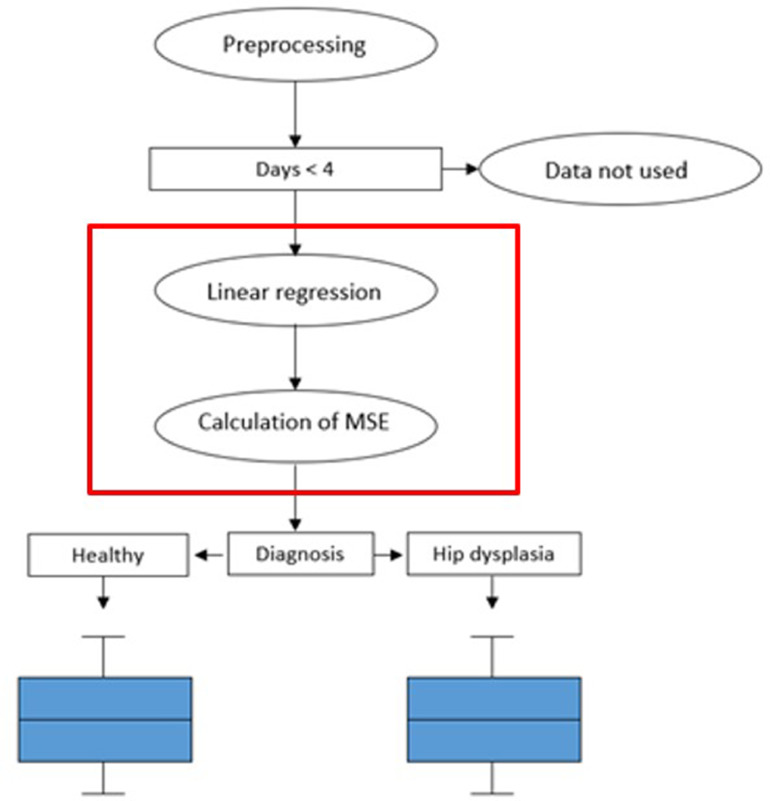
Schematic representation of the generation of the underlying data for Fig. 3, after preprocessing the data, sorting out the entries with too few measurement points, a linear regression is calculated for each feature. The residuals (mean square errors) are then calculated and dependent on the diagnosis grouped.

For the analysis, the measurements of the features were approximated for each dog individually over the entire period of the study using linear regression. The features were grouped based on the pathological side. For symmetry-based features, grouping was done by mirroring. Utilizing the linear regression, the mean square error (MSE) was calculated for each dog and subsequently assigned to either the "healthy" or the "HD" group based on the diagnosis made. The deviation of the measurements to the linear regression serves as the outcome measure for this study. By using this method, the influence of the dog breed factor can be neutralized. The deviations were displayed and compared in a box-whisker plot. The Shapiro-Wilks test was conducted for each feature to test the normal distribution of the mean square deviation. The unpaired *t*-test (independent *t*-test) with a significance level of 0.05 was used as the method for evaluating significance. The procedure is illustrated graphically in Fig. 2

**Fig. 3:**
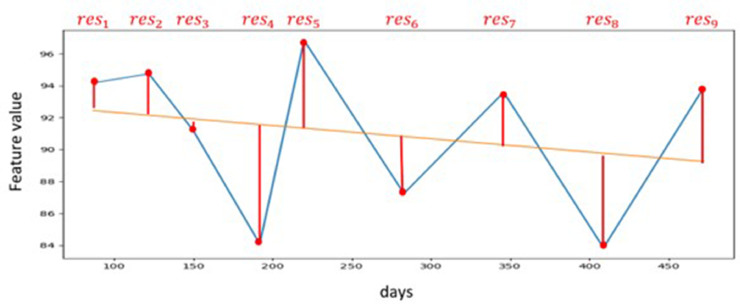
Visualisation of the calculation of the mean square error for a feature of a dog. X-axis: age of the dog on the day of the study, Y-axis: value of the calculated feature, orange: linear regression, red: deviations from the linear regression.

Fig. 3 illustrates an example of the variance calculations using linear regression. This step takes place within the highlighted area in red in Fig. 2. The X-axis displays the age of the dog on the day of the study, the Y-axis shows the value of the calculated feature. The red dots indicate the feature values determined on the examination day, while the blue line connects them. The linear regression of the feature values over the entire study area is depicted by the orange line. The red lines represent the deviation of the feature values from the linear regression (res_k_). The deviations are squared, totalled, then the mean value is calculated and assigned to the respective dog to serve as the outcome measure.

**Fig. 4:**
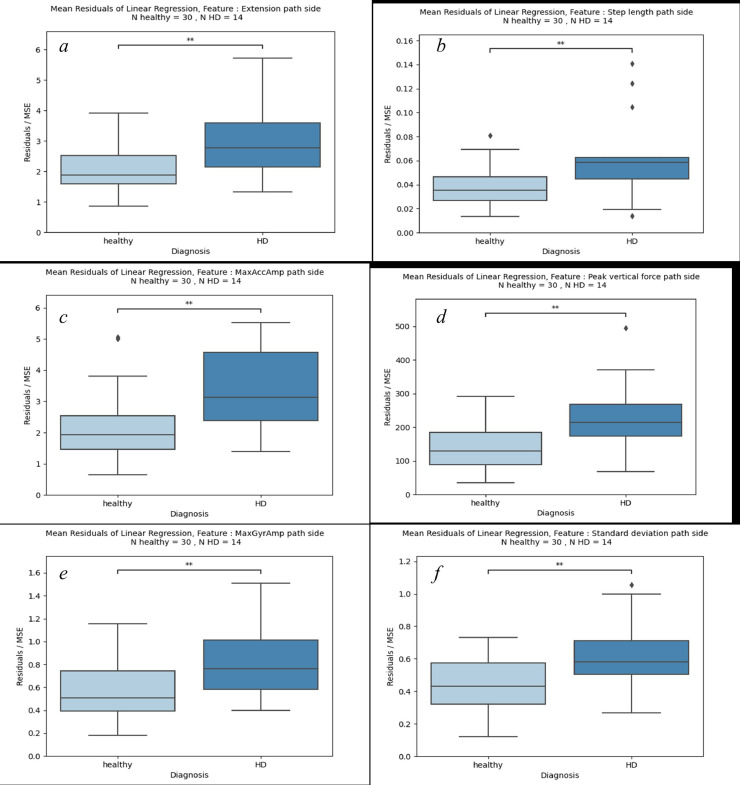
Variance of the linear regression of healthy dogs (left) and dogs diagnosed with HD (right) with calculation of significance via independent *t*-test. (*: *p* < 0.05, **: *p* < 0.01, ***: *p* < 0.001) The Y-axis shows the variances of the respective features: a) extension, b) stride length, c) maximum acceleration amplitude, d) maximum ventral acceleration, e) maximum gyroscope amplitude, f) standard deviation. All features refer to the side of the pathological limb.

## 3 Results

The data analysis revealed a remarkable difference in the mean value of the variance between the two groups for 13 features. Among these, the differences are highly remarkable (*p* < 0.01) for the 6 features directly affecting the pathological limb. The group of dogs diagnosed with HD showed a higher variance in gait pattern across all features throughout the whole study duration. The description of the features can be found in Table 3:

**Table 3:** Description of the analysed features.

**Feature**	**Description**
Extension	Average extension value from gyroscope
Step length	Length of one step
MaxAccAmp	Maximum Amplitude of Acceleration
Ventral acceleration	Peak acceleration in ground direction
MaxGyrAmp	Maximum amplitude of gyroscope
Standard deviation	Standard deviation of acceleration
Extension Symmetry Femur L - Humerus R	Symmetry of Extension of left femur to right humerus
MaxAccAmp Symmetry Femur R - Femur L	Symmetry of maximal acceleration amplitude of right femur to left femur
MaxAccAmp Symmetry Femur R - Humerus L	Symmetry of maximal acceleration amplitude of right femur to left humerus
MaxAccAmp Symmetry Femur L - Humerus R	Symmetry of maximal acceleration amplitude of left femur to right humerus
Normalised step length Symmetry Femur R - Humerus L	Symmetry of normalised step length of right femur to left humerus, normalised step length = step length divided by height
Step length Symmetry Femur R - Humerus L	Symmetry of step length of right femur to left humerus
Symmetry PR Lumbar	Symmetry of right hip extremity to Lumbar

In Fig. 4a–f six features affecting the pathological extremity are illustrated. In each comparison, the total number of individuals is *N* = 44. The 30 healthy dogs on the left side and 14 HD diagnosed dogs on the right side. The Y-axis represents the variances of the linear regression across the study period for the respective features. Across all features, the mean variance of the group diagnosed with HD is significantly higher than that of the healthy dogs (*p* < 0.01). The corresponding p-values are shown in Table 4.

**Table 4:** Associated p-values, mean values and standard deviations for Fig. 4.

**Feature**	***p*-value**	**Mean / Std healthy**	**Mean / Std HD**
Extension	3.1 × 10 ^−3^	2.03 / 0.74	2.91 / 1.10
Step length	3.3 × 10^–3^	0.03 / 0.01	0.06 / 0.03
MaxAccAmp	2.2 × 10^–3^	2.16 / 1.07	3.41 / 1.31
Peak vertical force	3.8 × 10^–3^	144.39 / 69.52	230.61 / 107.51
MaxGyrAmp	6.7 × 10^–3^	0.57 / 0.25	0.83 / 0.32
Standard deviation	5.2 × 10^–3^	0.43 / 0.16	0.61 / 0.22

Table 5 shows additional features with remarkable differences, along with their p-values, means and standard deviations. For the features listed, the mean variance values of the dogs diagnosed with HD proved to be higher as well.

**Table 5:** Further features with significant differences between the groups with associated *P*-values, means and standard deviations.

**Feature**	***p*-value**	**Mean / Std Healthy**	**Mean / Std HD**
Extension Symmetry Femur L - Humerus R	1.0 × 10^–2^	12.06 / 4.41	16.86 / 7.07
MaxAccAmp Symmetry Femur R - Femur L	4.4 × 10^–2^	3.95 / 1.25	5.19 / 2.06
MaxAccAmp Symmetry Femur R - Humerus L	9.9 × 10^–2^	10.40 / 4.4	14.34 / 4.42
MaxAccAmp Symmetry Femur L - Humerus R	1.4 × 10^–2^	9.58 / 3.61	13.02 / 4.48
Normalised step length Symmetry Femur R - Humerus L	1.0 × 10^–2^	3.26 / 2.72	8.60/ 9.93
Step length Symmetry Femur R - Humerus L	1.0 × 10^–2^	3.26 / 2.72	8.6 / 9.93
Symmetry PR Lumbar	1.1 × 10^–2^	2.21 / 0.90	2.98 / 1.13

## 4 Discussion

In this study, we observed thirteen features, showing remarkable differences between the healthy dogs and the dogs that have developed hip dysplasia. These form a robust foundation for further analysis (Fig. 4a–f). The gait features observed in the extremities diagnosed with hip dysplasia (HD) exhibited a notably higher variance compared to those of healthy dogs. Dogs that developed HD during the study period revealed an unstable gait pattern throughout their growth phase, as evidenced not only in the graphical representations but also in additional gait features detailed in Table 5. This observation indicates that the difference in the development of features between healthy dogs and those prone to HD lies in the variance observed over the growth period. Essentially, dogs developing HD exhibit a more unsteady gait pattern during their growth phase.

The same analysis, limited to measurements up to the 24th week of the growth phase, revealed a significantly higher variance in dogs suffering from HD compared to the overall measurement series. However, these results were not considered due to the limited number of dogs and measurement days, rendering them unrepresentative. This aspect presents a potential option for future investigations, suggesting the need for more examination days during the primary growth phase and further refinement of the automatic feature generation algorithm to minimize data loss during data preprocessing.

### 4.1 Enhancing the study

Enhancing the validity of data evaluation and statistical analyses can be achieved through several ways. An increased sample size, including a diverse range of breeds, and leveraging AI and deep learning data models are anticipated to provide a clearer overall picture. Subsequent steps involve implementing a method based on magnetometer and inertial sensors data fusion to enhance gait phase detection, step counting, and 2D/3D kinematic modelling. Improvements in the measurement process and the analytical algorithm can further reduce data exclusion, potentially enabling preventive classification with the use of inertial measurement units (IMUs). Additionally, neural networks for classification and the projection of IMU data onto a biomechanical model are considered promising approaches to enhance the opportunity of early diagnosis. To strengthen the robustness of the present study, a combination of the aforementioned procedures would be appropriate.

### 4.2 Broaden the scope

While this study focused on hip joint dysplasia, it is imperative to extend investigations to other diseases affecting puppies and young dogs, such as elbow dysplasia, osteochondrosis dissecans, and panosteitis. These polygenetic and multifactorial diseases lead to altered gait patterns, overloaded movements and thus presumably result in variance in the movement pattern. A comprehensive understanding of these pathologies, including their timing of development and the manifestation of clinical signs, could help to take preventive measures.

One notable preventive measure for incipient hip dysplasia is juvenile pubic symphysiodesis (JPS). Optimized early diagnosis in puppies, typically between 16 and 19 weeks old, offers an effective countermeasure against developing hip joint dysplasia, as highlighted in the publications by Dueland et al. (2010); Klever et al. (2020).

## Ethical statement

This manuscript matches the principles of the Committee on Publication Ethics. Furthermore, the involved use of animal subjects is according with the NC3Rs ARRIVE Gudelines.

## CRediT authorship contribution statement

**P. Blättler:** Resources, Methodology, Investigation. **M. Altermatt:** Writing – review & editing. **M. Röhrich:** Writing – review & editing. **N. Grütter:** Writing – review & editing, Writing – original draft, Methodology, Investigation, Formal analysis, Conceptualization.

## Declaration of competing interest

One author is respectively co-founder and shareholder of 4DVets AG, the manufacturer of the discussed system. All authors worked together on a research project financed by Orthovet, Fasanenstrasse 13, CH-4402 Frenkendorf.
